# Anticholinergics: A potential option for preventing posttraumatic epilepsy

**DOI:** 10.3389/fnins.2022.1100256

**Published:** 2023-02-24

**Authors:** Viviam Sanabria, Simone Romariz, Matheus Braga, Maira Licia Foresti, Maria da Graça Naffah-Mazzacoratti, Luiz Eugênio Mello, Beatriz M. Longo

**Affiliations:** ^1^Department of Physiology, Universidade Federal de São Paulo, São Paulo, Brazil; ^2^Instituto D’Or de Pesquisa e Ensino, São Paulo, Brazil

**Keywords:** traumatic brain injury, epileptogenesis, prevention, pharmacological treatment, anticholinergics

## Abstract

Interest in the use of anticholinergics to prevent the development of epilepsy after traumatic brain injury (TBI) has grown since recent basic studies have shown their effectiveness in modifying the epileptogenic process. These studies demonstrated that treatment with anticholinergics, in the acute phase after brain injury, decreases seizure frequency, and severity, and the number of spontaneous recurrent seizures (SRS). Therefore, anticholinergics may reduce the risk of developing posttraumatic epilepsy (PTE). In this brief review, we summarize the role of the cholinergic system in epilepsy and the key findings from using anticholinergic drugs to prevent PTE in animal models and new clinical trial protocols. Furthermore, we discuss why treatment with anticholinergics is more likely to prevent PTE than treatment for other epilepsies.

## 1. Introduction

Traumatic brain injury (TBI) is an interruption in normal brain function when a sudden force (i.e., rapid acceleration or deceleration of the brain, direct impact or injury due to explosions, or penetrating head injury) damages the brain (Carteri and da Silva, 2021). TBI has several causes, including traffic accidents, sports injuries, and falls (de Almeida et al., 2016). TBI is associated with long-term disability, neurological disorders, and early mortality, constituting a public health problem with socioeconomic burden in low- and middle-income countries (Arciniegas et al., 2002; de Almeida et al., 2016; Maas et al., 2017).

One of the main consequences of TBI is posttraumatic epilepsy (PTE), which refers to the development of epilepsy after brain injury. According to the International League Against Epilepsy, epilepsy is considered in the following conditions: one unprovoked seizure and probability of recurrent seizure risk over 60%, a diagnosis of epilepsy syndrome, and two or more seizures occurring more than 24 h apart (Fisher et al., 2014).

Epileptogenesis in PTE is a continuous process in which a normal brain shows hypersynchronous excitability after a head injury (for review, see Golub and Reddy, 2022). After brain injury, seizures are categorized as immediate (≤24 h post-TBI), early (≤7 days), or late (>7 days) (Jennett, 1969). Trauma severity is correlated with the risk of epileptogenesis, with a 2.1% risk in mild, 4.2% in moderate, and 16.7% in severe cases of TBI (Annegers et al., 1998). The risk of developing epilepsy in patients with TBI is 29-fold higher than in the general population (Lowenstein, 2009), with TBI causing 10–20% of all symptomatic epilepsies (Annegers et al., 1998). Moreover, follow-up studies have indicated that PTE is diagnosed in approximately 80% of TBI cases within 2 years (Haltiner et al., 1997). Critical risk factors for PTE include intracranial hematoma, unconsciousness at hospital admission, chronic alcoholism, age ≥ 65 years, injury severity, and skull fracture (Annegers et al., 1998).

Unfortunately, the cascade of changes that transforms the non-epileptic brain into one that generates spontaneous recurrent seizures (SRS) is not completely understood due to its complexity (for review, see Löscher et al., 2015); In fact, Löscher et al. (2015) proposed that there is no latent period, and that epileptogenesis can start immediately after brain injury, suggesting that antiepileptogenic therapies should be administered as soon as possible. Thus, PTE is not yet preventable. Although brain alterations are correlated with brain injury severity, they are not necessarily involved in epileptogenesis. Moreover, not all individuals develop epilepsy after an acute brain injury, probably due to the endogenous repair response, absence of risk factors, or low insult severity (Pignataro et al., 2008).

One significant research benchmark from the U.S. National Institute of Neurological Disorders and Stroke (NINDS) has been focusing on preventing the occurrence of epilepsy and its progression (National Institute of Neurological Disorders and Stroke, 2022). Therefore, developing novel therapies for preventing the development and progression of epilepsy is vital in at-risk patients.

A prospective window of opportunity to prevent, intervene, and stop the development of PTE is the latent period between brain injury and SRS onset; a period that can vary from months to years in humans (French et al., 1993) and from days to weeks in rodent models (Mello et al., 1993; Cavalheiro, 1995). Neurochemical interventions during this period may modify focal development (Cavalheiro et al., 1994). This concept has led to various experimental studies, and some clinical trials evaluated whether prolonged administration of anti-seizure drugs after a brain injury can prevent or modify epilepsy development (for review, see Löscher et al., 2015). However, no compound test has been advanced into clinical practice (Saletti et al., 2019).

To date, the crucial roles of the glutamatergic and GABAergic systems in the expression and suppression of seizures and the development of epileptic conditions have been widely investigated in preclinical and clinical studies due to the classical mechanism of imbalance between excitation and inhibition. Nonetheless, studies focusing on the involvement of the cholinergic system in epileptic conditions have raised some interest, with recent studies showing the effectiveness of anticholinergics in modifying the epileptogenic process (Bittencourt et al., 2017; Benassi et al., 2021; Meller et al., 2021).

In this review, we discuss the role of the cholinergic system in epilepsy and the use of anticholinergic drugs to treat PTE in animal models and clinical trials. Additionally, we discuss why PTE is more likely to be prevented than other types of epilepsy. Further, we considered that administering a selective cholinergic antagonist immediately after a brain injury attenuates excitotoxicity and consequently inhibits the development of PTE by interfering with the epileptogenic process at a critical moment. This review aims to provide new insights based on what has been proposed regarding PTE treatment using an anticholinergic strategy.

## 2. Acetylcholine and the cholinergic system

Acetylcholine (ACh) is an endogenous neurotransmitter synthesized from choline and acetyl-CoA in the cytoplasm of nerve terminals *via* the acetyltransferase enzyme and subsequently transported into vesicles (for review, see Zaagsma and Meurs, 2006). After release, ACh binds to two types of membrane proteins: metabotropic muscarinic receptors, which signal through either Gq proteins (M1, M3, and M5 subtypes) that activate phospholipase C, Gi/o proteins that are negatively related to adenylate cyclase (M2 and M4 subtypes), and ionotropic nicotinic receptors, which function as non-selective, excitatory channels. Within the synapse, ACh is converted back into choline and acetic acid by the acetylcholinesterase enzyme. Finally, choline is reuptaken by choline transporters (Pavel et al., 2006).

In the brain, ACh is broadly distributed; all regions of the forebrain, midbrain, and brainstem contain cholinergic neurons. Projections originating in the medial septum and diagonal band of Broca’s canal *via* the pre-commissural branch of the fimbria-fornix pathway constitute the main cholinergic inputs of the hippocampus, providing direct information to both principal neurons and interneurons (for a review, see Drever et al., 2011). In the substantia innominata of the basal forebrain, the nucleus basalis of Meynert has neurons projecting throughout the cortex and amygdala (Mesulam et al., 1983). Moreover, all the peripheral and central nervous systems cholinergic neurons use ACh as their neurotransmitter (pre- and post-ganglionic parasympathetic neurons and all pre-ganglionic sympathetic neurons) (for review, see Wang et al., 2021).

Cholinergic neurons are implicated in several crucial physiological processes, such as attention, learning, memory, and stress response (Wang et al., 2021). Additionally, ACh functions as a neuromodulator by coordinating the firing of neuronal clusters, increasing or decreasing synaptic dynamics, neuroplasticity, and spine density, increasing the release of growth factors, and hippocampal neurogenesis. Cognitive processes improve through an increase in cholinergic synapses, and the degeneration of central cholinergic neurons impairs memory (for review, see Maurer and Williams, 2017).

Due to its critical role in cognitive processes, ACh is highlighted as an essential factor in several diseases, such as depression (Saricicek et al., 2012), Alzheimer’s (Wilcock et al., 1982), Parkinson’s (Bohnen and Albin, 2011), and Huntington’s diseases (Ferrante et al., 1987) which show ACh denervation and imbalance.

Considering these findings, anticholinergic drugs that block and decrease the activity of ACh synapses in the central nervous systems have been prescribed for Parkinson’s, amyotrophic lateral sclerosis, and depression (Hori et al., 2015; McGeachan et al., 2017; López-Álvarez et al., 2019). Anticholinergic medications achieve the intended therapeutic effect by competing one-for-one with ACh, mostly at muscarinic receptors, modulating the receptor’s affinity, and reducing the physiological response by avoiding neuronal injury and subsequent inflammation (for review, see Scarr, 2012). However, various adverse effects on the central and peripheral nervous systems have been described with anticholinergics, including cognitive impairment and blurred vision (for review, see López-Álvarez et al., 2019).

## 3. PTE and cholinergic innervation

In PTE, the hippocampus presents a persistent reactive gliosis and a consistent neuronal loss in the hilar, CA1, and CA2 regions (Diaz-Arrastia et al., 2000). Although this structure commonly presents hippocampal sclerosis, other structures also undergo neurodegeneration; the brain stem, cerebellum, basal forebrain, and selected thalamic nuclei are also damaged (Maxwell et al., 2004). However, no studies have investigated neuropathological differences between the hippocampus and other structures in patients with TBI who did not develop PTE and those who developed PTE (Pitkänen and McIntosh, 2006). Most patients with PTE have seizure foci in the temporal lobe (56% of the cases), followed by the frontal lobe (36%), parietal lobe (5%), and occipital lobe (3%) (Hudak et al., 2004). This high percentage of patients with PTE with temporal lobe lesions reflects the relevance of the entorhinal cortex-hippocampus complex as these regions are frequently the site of origin of seizure activity, and both areas are enriched with cholinergic innervation (for review, see Friedman et al., 2007).

**FIGURE 1:**
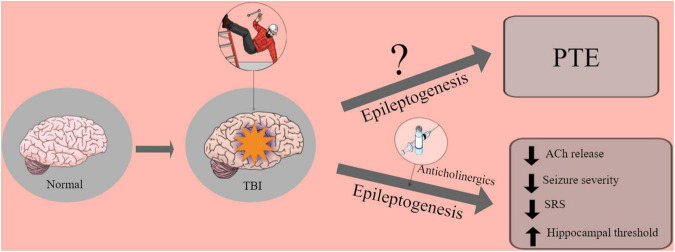
Graphic abstract of the role of anticholinergics drugs in the brain.

Early after TBI, the cholinergic system activates a large number of muscarinic receptors leading to an immediate massive release of ACh (increasing 49%), which can lead to status epilepticus (SE) (Turski et al., 1983; Saija et al., 1988; Shin and Dixon, 2015). However, in the chronic periods after TBI, cholinergic hypoactivity occurs. It has been speculated that cholinergic activity may be reduced due to cholinergic neuron dysfunction caused by functional deficits or neuronal loss (Östberg et al., 2011) (see Figure 1).

The mechanism underlying ACh hyperexcitability has been demonstrated by a stretch injury system applied to cerebral axons *in vitro*, inducing abnormal sodium influx through mechanically sensitive Na^+^ channels leading to increased intra-axonal calcium *via* the opening of voltage-gated calcium channels and reversal of the Na^+^-Ca^2+^ exchanger (Wolf et al., 2001; Arciniegas, 2003). Consequently, deformed axons were depolarized, and action potentials were propagated, leading to an excessive release of ACh or glutamate, excitotoxic to presynaptic and postsynaptic targets. Glutamate excitotoxicity may lead to excessive ACh and neuronal injury secondary release (Palmer et al., 1993). Moreover, TBI injury is related to blood-brain barrier damage, glial cell activation, and the release of pro- and anti-inflammatory cytokines (Korn et al., 2005; Pan et al., 2012). These pathological processes lead to excessive excitation of neurons and ultimately drive PTE.

In laboratory rats exposed to head trauma, the levels of ACh increased by 33% after TBI (Saija et al., 1988). Additionally, a study by Hillert et al. (2014) assessed changes in ACh release in the hippocampus before, during, and after SE *via* microdialysis in rats exposed to the lithium-pilocarpine model. Administration of a subconvulsant dose of pilocarpine increased hippocampal ACh release by six-fold, mainly in the striatum and hippocampus (Hillert et al., 2014).

Another study by Benassi et al. (2021) tested the relevance of the cholinergic system in epileptogenesis through a sophisticated experiment in which 192 IgG-saporin (a toxin that selectively kills cholinergic basal forebrain neurons) was injected into the right lateral ventricle, followed by induction of SE with subconvulsant doses of either pilocarpine or kainic acid. The results showed that 192 IgG-saporin-treated animals with a complete lesion of the cholinergic basal forebrain, did not exhibit SRS over 10 weeks after SE. Thus, this study showed that basal forebrain ACh release was relevant in epileptogenesis as a modulator of hippocampal transmission (Benassi et al., 2021).

These studies indicate that cholinergic innervation is essential for the onset and propagation of epileptic seizures, seizure-induced brain damage, and epileptogenesis, irrespective of the mechanism of seizure induction, at least in rodent models of epilepsy (for review, see Friedman et al., 2007).

## 4. Anticholinergics and insights from animal models of epilepsy

Reliable animal models are essential to understand how the progression of trauma contributes to epileptogenesis in PTE and to help develop antiepileptogenic treatment. Fluid percussion injury (FPI) and controlled cortical impact (CCI) are the most extensively used PTE models. In the FPI model, a precise pressure pulse is distinctly delivered in the animal dura mater using a closed hydraulic system (Pitkänen and McIntosh, 2006; Alder et al., 2011), followed by a chemoconvulsant as a second hit. In contrast, the CCI model produces brain injury using a pneumatic or electromagnetic impact to compress the exposed brain, causing brain injury (Scheff et al., 1997).

Along with the PTE models, conventional models, such as pilocarpine, kindling, pentylenetetrazol, and genetics models, have been extensively used to study epilepsy and investigate the prevention of this disease (Löscher, 2011; Meller et al., 2021). These animal models also play a crucial role in identifying cellular and epileptogenic mechanisms that are key to the discovery of novel treatments for PTE.

Many anticholinergics have been investigated and proposed as potential treatments for PTE using different animal models of epilepsy. Scopolamine and biperiden are the most well-known of these drugs (Eşkazan et al., 1999; Pereira et al., 2005; Bittencourt et al., 2017). Till now, no studies have evaluated the effects of cholinergic drugs in animal PTE models. Therefore, this section revisits the principal findings highlighting anticholinergics as a promising strategy to prevent PTE (see Table 1).

**TABLE 1:** Summary of laboratory studies using anticholinergics with animal models for posttraumatic epilepsy (PTE).

Anticholinergic	Epilepsy model	Protocol	Findings	References
Scopolamine	FPI	Experiment: pre-injury treatment with scopolamine. Adult male Sprague–Dawley rats were assigned to one of four groups: scopolamine 0.1, 1.0, or 10.0 mg/kg, i.p., or an equivalent volume of saline. Drugs were administered 15 min before the injury. Experiment: post-injury treatment with scopolamine. Adult male Sprague–Dawley rats were assigned to one of two groups: scopolamine (1.0 mg/kg, i.p.) or an equivalent volume of saline. Drugs were administered 30 s after injury.	-Scopolamine administrated before and after injury significantly attenuated the duration of suppression of behavioral responses. -Prior administration of scopolamine reduces the incidence of acute seizures and the associated prolonged apnea. -Pretreatment with a 1.0 mg/kg dose of scopolamine reduced mortality by 50%.	Lyeth et al., 1988
	CCI	Adult male Sprague–Dawley rats were trained with the Morris water maze. After five consecutive days of training, injured and uninjured rats were injected with scopolamine (1 mg/kg, i.p.). The drug was administered 15 min before testing in the maze. The next day rats were retested without scopolamine.	-Examined the effects of scopolamine on spatial memory in injured and uninjured rats and determined that when scopolamine was administrated, injured rats showed longer latencies to find the Water-Maze platform compared to sham-injured controls.	Dixon et al., 1994
	CCI	Adult male Sprague–Dawley rats were anesthetized and underwent two craniotomies before receiving a cortical impact through the right craniotomy. Fourteen days, post-injury, rats were surgically prepared for measuring extracellular ACh. Samples were collected every 20 min. After an equilibration period, scopolamine (1 mg/kg, i.p.) was injected. ACh levels were measured using an HPLC system.	-Controlled cortical impact decreases scopolamine-evoked release of ACh in freely moving rats at 14 days following injury.	Dixon et al., 1996
	Pilocarpine	Adult male Wistar EPM-1 rats were injected with pilocarpine (320–350 mg/kg, i.p.) and assigned to one of three groups according to scopolamine doses: 1 or 2 mg/kg, i.p., or an equivalent volume of saline. Scopolamine was injected 2 h after the onset of SE and terminated 17 days later.	-Animals treated with scopolamine had fewer SRS than animals without treatment.	Pereira et al., 2005
	Pilocarpine or kainic acid	Adult male Wistar rats were induced wiyh injections of pilocarpine (350 mg/kg, i.p.) or kainic acid. Approximately 30 min after pilocarpine and 2 h after kainate injection, most animals had reached SE. Animals were assigned to one of four groups according to scopolamine doses: 1, 2, 8 mg/kg, i.p., or an equivalent volume of saline. Scopolamine injections initiated 2 h after the onset of SE. Subsequent injections were administered every 6 h for the following 3 days, followed by administration *via* an osmotic pump for an additional 14 days. Injections were terminated on the 17th day.	-Scopolamine treatment decreased SRS when it was given in lower doses.	Benassi et al., 2021
	Lithium-pilocarpine	Adult female Sprague–Dawley rats were injected with lithium chloride (127 mg/kg, p.o.) 12–18 h before pilocarpine treatment. Pilocarpine was then administered at a bolus dose of 30 mg/kg, i.p. If needed this was followed by repeated i.p. injection of 10 mg/kg every 30 min until the onset of SE. Treatment with scopolamine (10 mg/kg i.p.) started 2 h after SE onset; the dose was divided by 4 mg/kg administered at 8 a.m. and 6 mg/kg at 6 p.m. over 17 days.	-Compared to the vehicle group, seizure frequency was significantly lower in the scopolamine group at both 8–10 and 24–26 weeks post-SE.	Meller et al., 2021
Pirenzepine	Kindling	Adult male Sprague–Dawley rats underwent bipolar nichrome stimulation and recording electrodes were implanted into the left and right basolateral amygdala and over the cortex. Amygdala stimulation was applied daily with an initial stimulus of 50 μA, and seizure intensity was graded according to Racine’s scale. In experiment 1, animals received saline or pirenzepine at doses of 10, 25, 50, and 100 nmol intracerebroventricularly 1 h before each electrical stimulation. In experiment 2, animals were kindled to the complete stage, and after a recovery period of 3–5 days, a dose of 50 nmol of pirenzepine was administered intracerebroventricularly to kindled animals.	-In experiment 1, the animals pretreated with 50 and 100 nmol doses failed to develop stages 4 or 5 of Racine. Suggesting a role for the M1 receptor in the kindling process. -In experiment 2, the seizure stage and after discharge duration were not affected by pirenzepine in fully kindled animals.	Eşkazan et al., 1999
Biperiden	Pilocarpine	Adult male Wistar rats were induced with injections of pilocarpine (320 mg/kg, i.p.). Biperiden was injected at 8 mg/kg i.p. every 8 h for 10 days, starting 3 h after SE onset.	-Biperiden treatment over 15 to 105 days diminished the frequency of spontaneous epileptic seizures.	Bittencourt et al., 2017
	Pilocarpine or kainic acid	Adult male Wistar rats were induced with injections of pilocarpine (350 mg/kg, i.p.) or kainic acid. Approximately 30 min after pilocarpine and 2 h after kainate injection, most animals had reached SE. Biperiden was injected at 8 mg/kg i.p. every 8 h (starting 3 or 6 h after SE onset) for 5, 10, or 20 days of biperiden administration.	-Animals treated with biperiden for only 5 days showed no effect, but when the treatment was expanded for 20 days, the latency of the first seizure increased, and SRS reduction was observed.	Benassi et al., 2021

FPI, fluid percussion injury; CCI, controlled cortical impact; SRS, spontaneous recurrent seizure; ACh, acetylcholine; i.p., intraperitoneal; p.o., oral administration; h, hours; min, minutes; s, seconds; μA, microampere.

In Lyeth et al. (1988) performed different experiments with scopolamine (a high-affinity muscarinic antagonist) to attenuate transient behavioral suppression and physiological responses in rats that had experienced brain injury through the FPI model. They observed that transient behavioral suppression produced by moderate brain injury could be mitigated by prior administration of scopolamine (Lyeth et al., 1988).

Accordingly, Dixon et al. (1994) examined the effects of scopolamine on spatial memory by the Morris water maze test in uninjured and injured rats using the CCI model (see Table 1). Interestingly, when scopolamine was administered, the injured rats demonstrated longer latencies to find the hidden platform than the controls (which may be due to the adverse effects of the anticholinergic drug). This result led them to speculate that changes in cholinergic neurotransmission by receptor blockade rendered this system more vulnerable to the impact (Dixon et al., 1994). Similarly, 14 days post-brain injury, scopolamine evokes less ACh in neocortex and hippocampus of injured animals, which may propose a posttraumatic cognitive deficit (Dixon et al., 1996). However, *in vivo* study did not demonstrate that the site of action of scopolamine is presynaptic. Thus, muscarinic antagonists may block receptors located on the cell bodies of inhibitory interneurons, leading to increased brain ACh release at late TBI phase (Dixon et al., 1996).

Furthermore, a study by Pereira et al. (2005) evaluated the use of scopolamine after the onset of pilocarpine-induced SE. Although they did not observe any change in the behavioral characteristics of the ongoing SE, scopolamine was found to reduce SRS frequency by 50%, although it did not affect mossy fiber sprouting in the dentate gyrus. Therefore, scopolamine interferes with the epileptogenic process (Pereira et al., 2005). More importantly, this is the first report to show the effect of anticholinergic agents on the epileptogenic process, in which the tested compound was injected hours after the insult (not before).

Besides acting on acute provoked seizures, scopolamine has also shown encouraging results as an antiepileptogenic in two animal models of epilepsy: pilocarpine and kainic acid. More recently, the effects of scopolamine were also reported by Benassi et al. (2021), who validated different animal models of epilepsy and searched for the optimal dose for treatment (Benassi et al., 2021). Intriguingly, the results indicated that the lowest dose of scopolamine presented the best results in both models, considering the SRS occurrence. In contrast, higher-dose animals were indistinguishable from those that did not receive any scopolamine treatment in the SE group suggesting an inverted dose-effect curve (Benassi et al., 2021).

Another 2021 study by Meller et al. (2021) treated laboratory rats with scopolamine and compared it with vehicle control treatment (see Table 1). Scopolamine treatment during the latent period following lithium-pilocarpine-induced SE did not significantly reduce the number of rats with SRS 2 months post-SE. However, at 6 months post-SE, the number of rats with SRS was reduced considerably (>60%) (Meller et al., 2021), suggesting that prolonged treatment with scopolamine after lithium-pilocarpine-induced SE blocks muscarinic receptors during epileptogenesis and prevents hypersensitivity to ACh release (Meller et al., 2021).

Pirenzepine, a competitive M1 muscarinic, was also analyzed, and its administration was evaluated pre- and post-completely kindle laboratory rats. Anticholinergic effect was not observed in completely kindled animals but was involved in the epileptogenesis of the kindling model (Eşkazan et al., 1999).

Biperiden, another muscarinic antagonist widely prescribed for Parkinson’s disease, have also been investigated (Benassi et al., 2021). Bittencourt et al. (2017) performed an experiment in which rats were injected with pilocarpine and then administered biperiden (Table 1; Bittencourt et al., 2017). The authors concluded that biperiden treatment reduced the severity and number of SRS by elevating the threshold of hippocampal excitability (Bittencourt et al., 2017).

The duration of the blockade of cholinergic transmission necessary to suppress epileptogenesis was tested by Mello’s group. Biperiden was administered for 5, 10, or 20 days to determine the optimal treatment duration (Benassi et al., 2021). No effect was found in the group that received biperiden for only 5 days. However, the groups that received treatment for 10 and 20 days showed a significant increase in the latency for the first seizure and a substantial reduction in SRS frequency (Benassi et al., 2021). Additionally, animals treated with biperiden performed better in learning and memory tests after 3 months than those without treatment (Benassi et al., 2021). The authors proposed that biperiden decreased SRS incidence and may effectively reduce epilepsy after an insult.

Overall, essential differences between scopolamine and biperiden have been identified for their therapeutic applications in epileptic conditions. Scopolamine acts as a non-selective muscarinic antagonist that produces both peripheral and central antimuscarinic effects, such as sedative, antiemetic, and amnestic effects (Renner et al., 2005).

In contrast, biperiden is a relatively specific Ml antimuscarinic antagonist with weak peripheral anticholinergic action and with pronounced effects on the central nervous system, resulting in relevant properties for therapeutic application (DRUGBANK, 2022). Furthermore, biperiden is on the World Health Organization’s List of Essential Medicine (World Health Organization, 2021), making it an attractive potential antiepileptogenic treatment for PTE.

## 5. Clinical trials using anticholinergics drugs in patients with PTE

To date, no favorable results for avoiding epileptogenic processes have been obtained using anti-seizure drugs such as phenytoin, carbamazepine, or valproate. Their use is frequently recommended only during the first-week post-injury to suppress immediate and early seizures (Carney et al., 2017).

According to the ClinicalTrials.gov database, only six out of 12 active studies related to PTE are performing drug interventions. These include studies on levetiracetam (ClinicalTrials.gov Identifier: NCT01463033, a completed phase 2 study), [18F] DPA-714 (NCT03999164, ongoing only in phase 1), allopregnanolone (NCT01673828, completed phase 2), valproate sodium (NCT00004817, completed phase 3), and biperiden (NCT01048138, recruiting in phase 3; NCT04945213, not yet recruiting in phase 3).

Among these, biperiden is the only anticholinergic drug that has been investigated in a PTE clinical trial to evaluates its safety and efficacy. Although presenting dose- and concentration-dependent temporary declines in cognitive functioning (Bakker et al., 2021), biperiden was proved safe in a phase 2 study that tested its use after TBI, showing the protocol feasibility and possible prospective efficacy (Benassi et al., 2021; Foresti et al., 2022).

## 6. Why is PTE more likely to be prevented than other epilepsies?

As mentioned earlier, PTE is likely to develop after a TBI of great severity; however, PTE does not necessarily occur in all cases (Löscher, 2020). Furthermore, epilepsy after TBI is potentially preventable because posttraumatic seizures that follow this injury have a high incidence of presenting only after several years (Christensen, 2015). This period between the trauma event and the putative appearance of seizures, offers an open window; if treated correctly, it might stop the epileptogenesis process.

Therefore, considering the predicted risk of epileptogenesis after TBI (Pitkänen et al., 2016), prevention of primary brain injury is key to preventing PTE. This aspect differentiates PTE from other epilepsies in which the causes and development of the disease remain unknown until the first seizure. Furthermore, prevention is vital, as patients with PTE are frequently pharmacoresistant and usually unsuitable surgical candidates (Garga and Lowenstein, 2006).

## 7. Conclusions and perspectives

Posttraumatic epilepsy is a frequent consequence of TBI, depending on the trauma severity and internal and external risk factors. Interest in the cholinergic pathway for preventing PTE has recently grown because of the effectiveness of anticholinergics in modifying the epileptogenic process. Consequently, different anticholinergics have been studied in various laboratories. Scopolamine and biperiden have demonstrated promising results by reducing seizure severity and frequency, decreasing SRS, and increasing hippocampal threshold in different animal models. In particular, biperiden has drawn researchers’ attention and shown exciting results. To date, biperiden is the only anticholinergic drug studied in clinical trials and passed all safety and efficacy tests, raising expectations as the first antiepileptogenic drug. Additionally, considering the need to find effective strategies to prevent epileptic conditions, PTE seems potentially preventable compared to other types of epilepsies, since TBI has a latent period for interventions, thereby increasing the chance of a positive result. Future studies should clarify the anti-epileptogenic properties and overcome the challenge of preventing epileptic conditions caused by TBI.

## Author contributions

VS wrote the first draft of the manuscript. VS, BL, and MF contributed to the article’s literature search, analysis, and conception. SR, MB, MN-M, and LM contributed to the revision of the manuscript. All authors approved and read the submitted version.
